# Provings of Lac Caninum

**Published:** 1884-10

**Authors:** E. W. Berridge

**Affiliations:** London


### PROVINGS OF LAC CANINUM.

E. W. Berridge, M. D., London.

I. Dr. Arabella K., æt. twenty-four, dissolved two globules of *Lac caninum*$^{cm}$ (Fincke) in water and took a spoonful every half hour. I gave her this medicine to convince her of the power of high potencies to produce morbid conditions. Almost immediately after the first teaspoonful she had a dull, aching pain in right eyeball, accompanied by a feeling of dryness and discomfort, with tendency to lachrymation. The left eye was not affected. She continued the frequent doses, but no further effect was noticed.

II. June 4th, 1884, I gave Mrs. B., æt. twenty-nine, for dysmenorrhœa, *Lac caninum*$^{cm}$ (Fincke), a dose twice a day for fourteen days. It caused decided improvement, but was said to have caused toothache.

On inquiring for particulars, the following symptoms were sent to me August 6th: "It came on very soon after commencing the medicine. The pain used to come on quite suddenly at about 10.30 P. M. every night. If not before I went to bed, it would begin almost immediately on lying down or getting warm in bed, so that I was obliged to jump up and often walk about the room. The pain seemed to begin in a very much decayed right lower double tooth, but after a while *all* the teeth on right side would ache. It was relieved by the application of cold water or a dose of *Chamomilla*, which would often quiet it immediately. It continued for quite a month, coming on each night, and the teeth often feel tender and as if they were too long or too large still. It was often daylight before I could get any sleep at all. The pain was not very bad; it was a dull, gnawing sensation, as of a worm gnawing at the teeth—just enough to keep me wide awake. I have had the same kind of pain in face and teeth from over-fatigue or much worry, but never at a stated hour or lasting so many hours at a time, nor so many days in succession. It came on at the same time whether I had my last meal early or late or went without it. If I went to bed earlier and fell asleep, it would wake me up when the hour arrived."

